# IMPROVING MEDICATION MANAGEMENT FOR HOME-DWELLING OLDER ADULTS WITH COGNITIVE IMPAIRMENT: END-USER INPUT

**DOI:** 10.1093/geroni/igac059.2906

**Published:** 2022-12-20

**Authors:** Rana Zadeh, Cary Reid, Elaine Wethington, Sara Czaja, Elizabeth Capezuti, Wendy Shields, Deb Traunstein

**Affiliations:** Cornell University, Ithaca, New York, United States; Weill Cornell Medicine, Ithaca, New York, United States; Cornell, Ithaca, New York, United States; Weill Cornell Medicine, Ithaca, New York, United States; Hunter College of the CUNY (City University of New York), New York, New York, United States; Johns Hopkins University, Baltimore, Maryland, United States; Visiting Nurse Service of Ithaca and Tompkins County, Ithaca, New York, United States

## Abstract

HOPES™ Program is a joint community-university partnership, intended to build capacity for improving medication adherence and reducing medication mistakes, misuse, and abuse for community-dwelling older adults with reduced cognitive functioning (CWCs) in New York State (NYS). The goal of this program is to contribute to the health and safety of older adults, empower them and their caregivers to reduce medication non-adherence consequences and risks, and enable older adults to continue living at home while managing their health conditions. This program applied a collective user-centered approach to connect resources, identify and address barriers, and reach the most vulnerable groups. One aspect of our user-centered approach included a medication management and safety training module video that was adapted from the Medication Use Safety Training for Seniors™ (MUST for Seniors™) and underwent alpha and beta testing. A qualitative analysis was also conducted on input from home-care providers, CWCs, and their designated caregivers about the current practices, challenges, and solutions related to medication management. Our preliminary findings demonstrated a need for research on reducing the steep estimated national costs of medication non-adherence. Thus, a sub-team has developed a methodology to build an economic perspective on the scope and magnitude of interventions to enhance medication management practices for CWCs. A NYS-specific survey was also developed in close collaboration with providers, university partners, and state-level advocacy organizations according to our preliminary findings to document the state and magnitude of medication non-adherence, discern risk factors and determinants of non-adherence, and explore solutions and barriers.

